# Supplementary material for: Present and Future of Parkinson’s Disease in Spain: PARKINSON-2030 Delphi Project
Source: Brain Sci. 2021 Jul 31;11(8):1027. doi: 10.3390/brainsci11081027 (PMC8393421; doi:10.3390/brainsci11081027)
Supplement: Supplementary file 1 [file brainsci-11-01027-s001.zip › brainsci-1316744-supplementary.pdf]

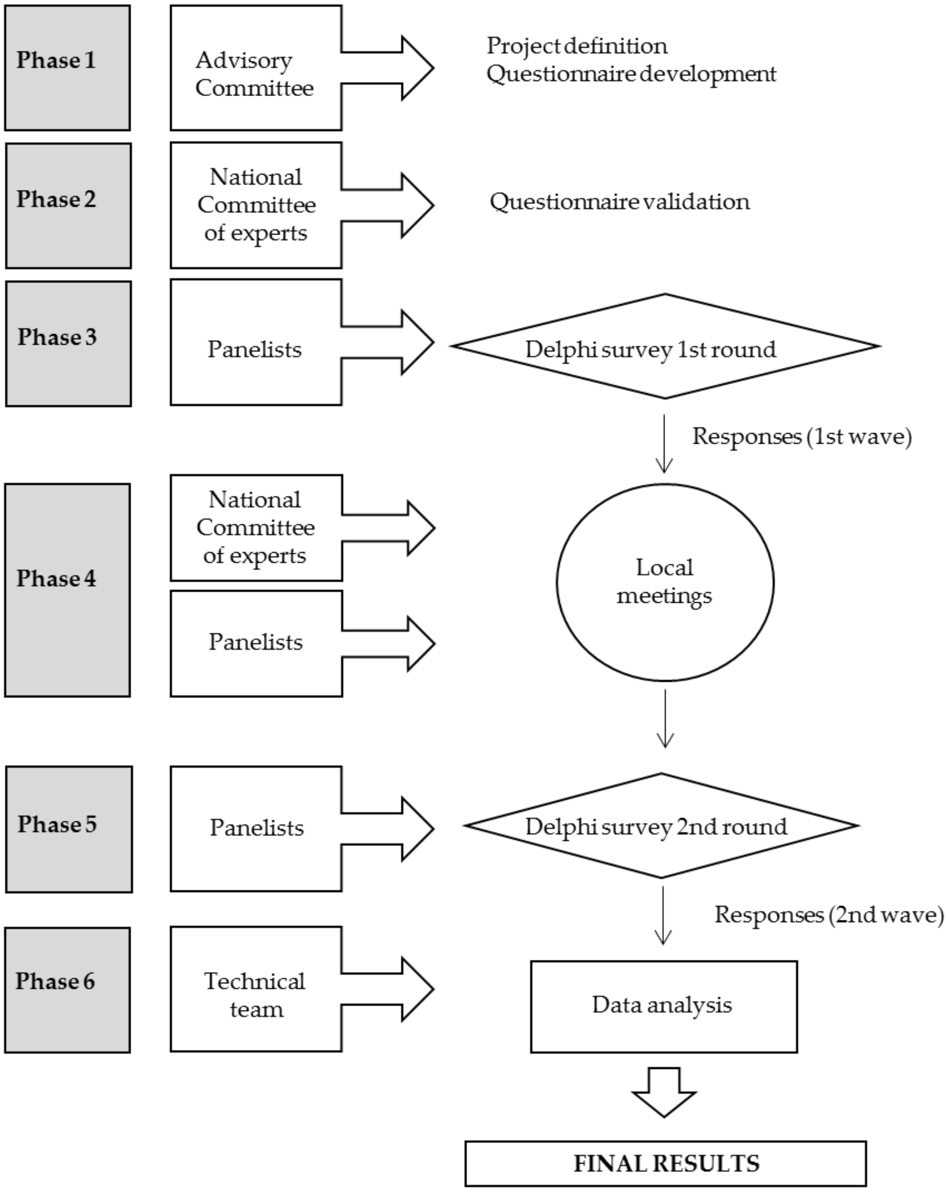

Figure S1. Flow chart of the study.

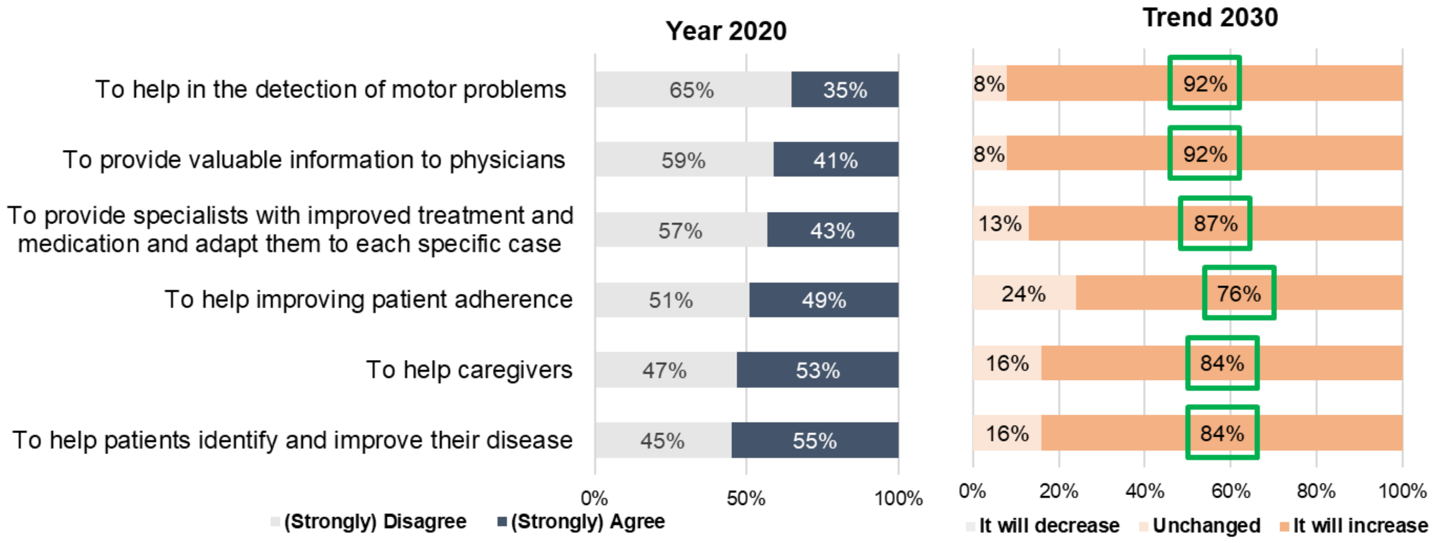

Figure S2. Contribution of new technologies for PD monitoring: present and future

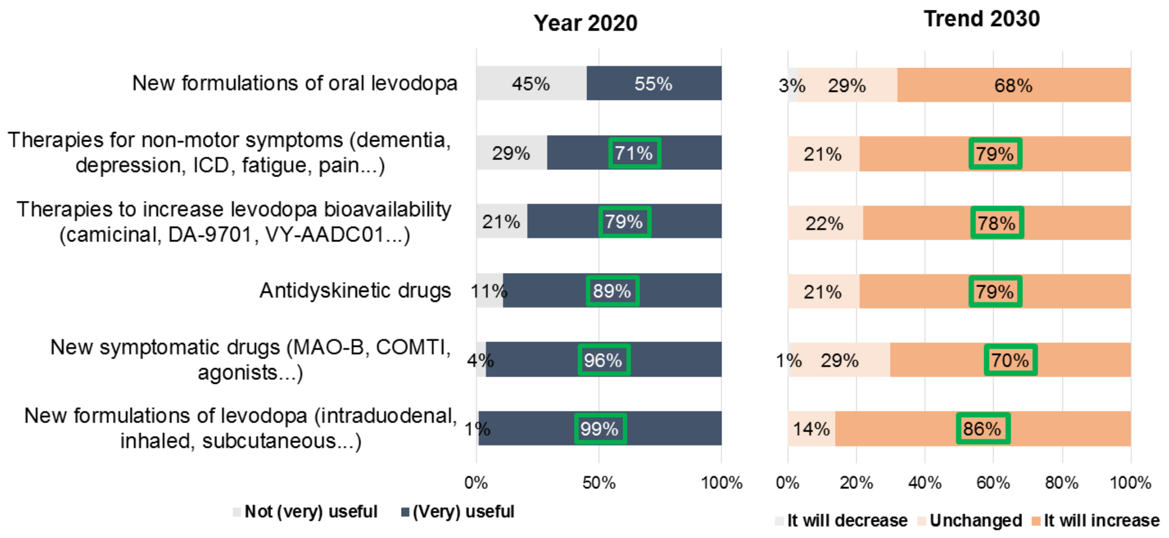

(**a**)

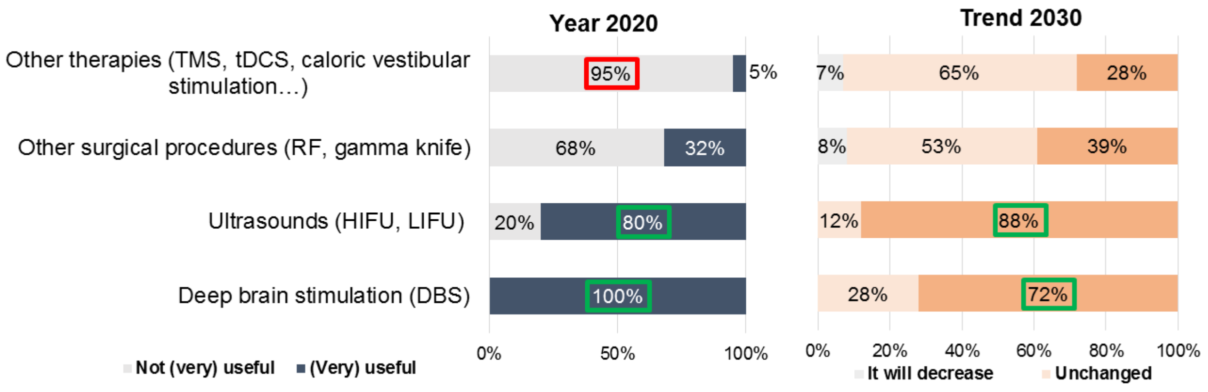

(**b**)

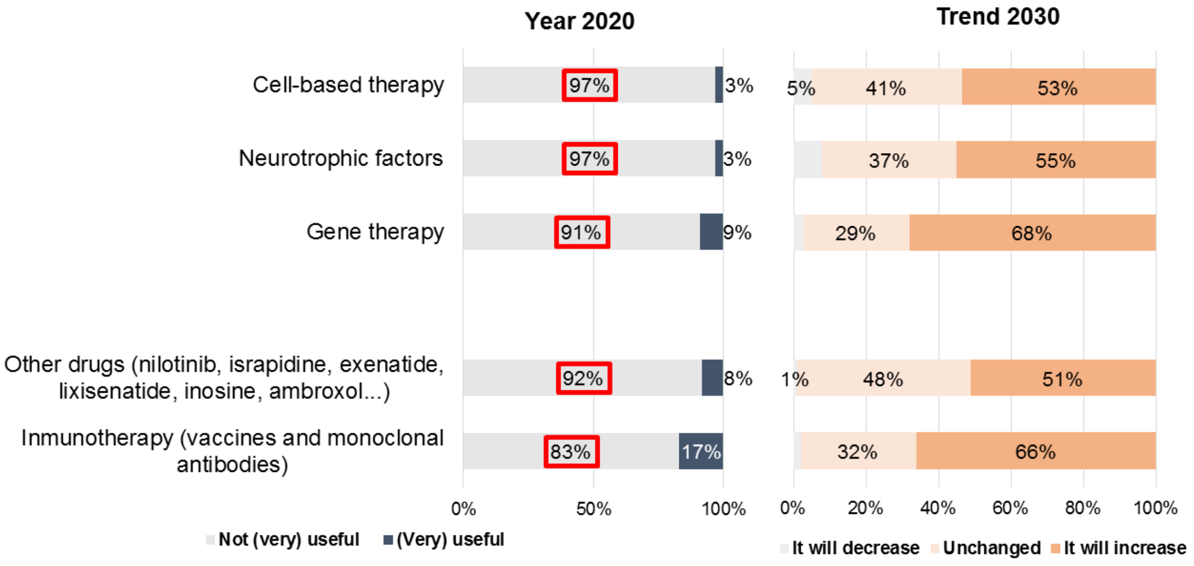

(**c**)

Figure S3. Investigational therapies of PD: present and future. (**a**) Perceived utility of pharmacological inves-tigational therapies. COMTI: catechol-O-methyltransferase inhibitors. ICD: impulse control disor-ders. MAO-B: monoaminooxidase B; (**b**) Perceived utility of non-pharmacological investigational therapies. HIFU: high-intensity focused ultrasound. LIFU: low-intensity focused ultrasound. RF: radiofrequency. tDCS: transcranial direct current stimulation. TMS: transcranial magnetic stimu-lation; (**c**) Perceived utility of disease-modifying and curative investigational therapies.

Table S1. List of participating panelists

Surname, Name
Alonso Cánovas, Araceli
Alonso Frech, Fernando
Álvarez Sauco, María
Aneiros Díaz, Ángel
Arias Rodríguez, Mónica
Ávila Rivera, Asunción
Berganzo Corrales, Koldo
Caballo Pons, Núria
Cabello de la Rosa, Juan Pablo
Cabo López, Iria
Campdelacreu Fumadó, Jaume
Carrillo García, Fátima María
Cimas Hernando, Icíar
Claramonte Clausell, Berta
Clavero Ibarra, Pedro
De la Casa Fages, Beatriz
Delgado Alvarado, Manuel
Delgado Ballestero, Tania
Errea Abad, José María
Escalante Arroyo, Sonia
Espinosa Rosso, Raúl
Fernández García, José Manuel
Fernández Moreno, María Del Carmen
Fernández Pajarín, Gustavo
Fernández Villalba, Emiliano
García Caldentey, Juan
García Fernández, Ciara
Garcia Trujillo, Lucía
Gómez Heredia, María José
Gómez Llopico, Roser
González Torres, Verónica
Gutiérrez García, Javier
Hernández Rubio, Lidia
Herreros Rodríguez, Jaime
Infante Ceberio, Jon
Ispierto González, Lourdes
Jaumà Classen, Serge
Jesús Maestre, Silvia
Llanero Luque, Marcos
López Ariztegui, Nuria
Lopez del Val, Luis Javier
Lorenzo Brito, Jesús Norelis
Martí Martínez, Silvia
Martín Bastida, Antonio

Martínez Fernández, Raúl
Moragues Benito, María Dolores
Muñoz Ruiz, Teresa
Ochoa Sepúlveda, Juan José
Olivares Romero, Jesús
Ordás Bandera, Carlos M.
Oropesa Ruiz, Juan Manuel
Paz González, José Manuel
Peña Llamas, Esteban
Perona Moratalla, Ana Belén
Pinel Ríos, Javier
Pont Sunyer, Claustre
Puente Periz, Víctor
Pueyo Morlans, Mercedes
Rodríguez Sanz, Ana
Rojo Sebastián, Ana
Ruiz Bajo, Blanca
Ruiz López, Marta
Ruiz Martínez, Javier
Sanchez Alonso, Pilar
Sanz Cartagena, Mª Pilar
Sierra Peña, María
Solano Vila, Berta
Suárez Muñoz, José A.
Suárez San Martín, María Esther
Turpin Fenoll, Laura
Valero Merino, Caridad
Vázquez Picón, Raquel
Vega Pérez, Jesús Manuel
Vela Desojo, Lydia
Vives Pastor, Bárbara

Table S2. Panelists' descriptions

Characteristic	n=75
Gender, n (%)	
Male	44 (58%)
Female	31 (42%)
Age, n (%)	
25-35 years	11 (15%)
36-45 years	32 (42%)
46-55 years	24 (32%)
>55 years	8 (11%)
Workplace*, n (%)	
Primary	3 (4%)
Secondary	17 (22%)
Tertiary	55 (74%)
Years of experience mean (SD)	14.3 (8.2)
Geographical distribution, n (%)	
Andalusia	14 (18.67%)
Aragon	2 (2.67%)
Asturias	3 (4.0%)
Balearic Islands	3 (4.0%)
Basque Country	5 (6.67%)
Cantabria	3 (4.0%)
Castilia La Mancha	3 (4.0%)
Catalonia	12 (16.0%)
Canary Islands	3 (4.0%)
Galicia	5 (6.67%)
Madrid, Community of	14 (18.67%)
Murcia, Region of	2 (2.67%)
Navarra	1 (1.33%)
Valencian Community	5 (6.67%)

* Primary (hospitals that carry out promotion and prevention activities, Local Health Plan activities, general medical consultations, general dentistry, clinical laboratory and low complexity imaging, hospitalization, emergency care and low complexity childbirth). Secondary (hospitals that carry out outpatient medical consultations, hospitalization and emergency care of basic specialties, medium complexity childbirth and cesarean section care, general and specialized dental care, nutrition, psychology, optometry and support therapies for functional rehabilitation). Tertiary (hospitals that provide medical care aimed at restoring health and rehabilitating patients referred by primary and secondary hospitals, who present conditions of high diagnostic and treatment complexity that involve one or more medical, surgical or medical-surgical specialties).
